# Supplementary material for: Characteristics and Pathogenicity of Discula theae-sinensis Isolated from Tea Plant (Camellia sinensis) and Interaction with Colletotrichum spp
Source: Plants (Basel). 2023 Sep 28;12(19):3427. doi: 10.3390/plants12193427 (PMC10574372; doi:10.3390/plants12193427)
Supplement: Supplementary file 1 [file plants-12-03427-s001.zip › plants-2567874-supplementary.pdf]

Table S1. Primers used in this study

Gene	Primer	Sequence (5’→3’)	Reference
CAL	CL1C	GAA TTC AAG GAG GCC TTC TC	[1]
	CL2C	CTT CTG CAT CAT GAG CTG GAC	[1]
GAPDH	GDF	GCC GTC AAC GAC CCC TTC ATT GA	[2]
	GDR	GGG TGG AGT CGT ACT TGA GCA TGT	[2]
ITS	ITS-1	CTT GGT CAT TTA GAG GAA GTA A	[3]
	ITS-4	TCC TCC GCT TAT TGA TAT GC	[4]
TUB2	T1	AAC ATG CGT GAG ATT GTA AGT	[5]
	Bt2b	ACC CTC AGT GTA GTG ACC CTT GGC	[6]
EF1	EF1-728F	CAT CGA GAA GTT CGA GAA GG	[7]
	EF1-986R	TAC TTG AAG GAA CCC TTA CC	[7]
RPB2	RPB2-P2F	GGA AGT GGT GGA GGA GTA CGA G	[8]
	RPB2-P3R	CTG GTT GTG GTC GGG GAA GGG	[8]
LSU	LROR	GTA CCC GCT GAA CTT AAG C	[9]
	LR7	TAC TAC CAC CAA GAT CT	[9]

References

1. Weir, B.S.; Johnston, P.R.; Damm, U. The *Colletotrichum gloeosporioides* species complex. *Stud. Mycol.* **2012**, *73*, 115–180.
2. Templeton, M.D.; Rikkerink, E.; Solon, S.L.; Crowhurst, R.N. Cloning and molecular characterization of the glyceraldehyde-3-phosphate dehydrogenase-encoding gene and cDNA from the plant pathogenic fungus *Glomerella cingulata*. *Gene* **1992**, *122*, 225–230.
3. Gardes, M.; Bruns, T.D. ITS primers with enhanced specificity for basidiomycetes—Application to the identification of mycorrhizae and rusts. *Mol. Ecol.* **1993**, *2*, 113–118.
4. White, T.; Bruns, T.; Lee, S.; Taylor, J. Amplification and direct sequencing of fungal ribosomal RNA genes for phylogenetics. *Pcr. Protocols*. **1990**, *18*, 315–322.
5. O'Donnell, K.; Cigelnik, E. Two divergent intragenomic rDNA ITS2 types within a monophyletic lineage of the fungus Fusarium are nonorthologous. *Mol. Phylogenet. Evol.* **1997**, *7*, 103–116.
6. Glass, N.L.; Donaldson, G.C. Development of primer sets designed for use with the PCR to amplify conserved genes from filamentous ascomycetes. *Appl. Environ. Microbiol* **1995**, *61*, 1323–1330.
7. Carbone, I.; Kohn, L.M. A method for designing primer sets for speciation studies in filamentous ascomycetes. *Mycologia* **1999**, *91*, 553–556.
8. Liu, Y.J.; Whelen, S.; Hall, B.D. Phylogenetic relationships among ascomycetes: Evidence from an RNA polymerse II subunit. *Mol. Biol. Evol.* **1999**, *16*, 1799–1808.
9. Vilgalys, R.; Hester, M. Rapid genetic identification and mapping of enzymatically amplified ribosomal DNA from several Cryptococcus species. *J. Bacteriol.* **1990**, *172*, 4238–4246.

Table S2. Isolates *Diaporthales* spp. studied and GenBank accession numbers of the generated sequences

Species	Accession number	GenBank accession			
		ITS	LSU	EF1	RPB2
Discula theae-sinensis	MAFF238240		AB511919		
	MAFF238241		AB511920		
	MAFF238242		AB511921		
	MAFF238243		AB511922		
	DX1	ON453684	ON340629	ON366583	ON568236
	DX2	ON453700	ON340645	ON366599	ON568247
	DX3	ON453689	ON340634	ON366588	ON568240
	DX4	ON453685	ON340630	ON366584	ON568237
	DX5	ON453708	ON340654	ON366608	ON568254
	DX6	ON453691	ON340636	ON366590	-
	DX7	ON453688	ON340633	ON366587	ON568239
	DX8	ON453699	ON340644	ON366598	ON568246
	DX9	ON453686	ON340631	ON366585	ON568238
	DX10	ON453687	ON340632	ON366586	-
	DX11	ON453723	ON340669	ON366623	ON568266
	DX12	ON453738	ON340688	ON366642	ON568282
	DX13	ON453712	ON340658	ON366612	ON568258
	DX14	ON453741	ON340691	ON366645	ON568285
	DX15	ON453740	ON340690	ON366644	ON568284
	DX16	ON453739	ON340689	ON366643	ON568283
	DX23	ON453704	ON340649	ON366603	ON568250
	DX24	ON453711	ON340657	ON366611	ON568257
	DX25	ON453709	ON340655	ON366609	ON568255
	DX26	ON453702	ON340647	ON366601	ON568249
	DX27	ON453710	ON340656	ON366610	ON568256
	DX28	ON453713	ON340659	ON366613	ON568259
	DX29	ON453725	ON340671	ON366625	-
	DX30	ON453737	ON340683	ON366637	ON568277
	DX31	ON453714	ON340660	ON366614	ON568260
	DX32	ON453721	ON340667	ON366621	ON568264
	DX33	ON453724	ON340670	ON366624	ON568267
	DX34	ON453693	ON340638	ON366592	ON568242
	DX35	ON453703	ON340648	ON366602	-
	DX36	ON453705	ON340650	ON366604	ON568251
	DX37	ON453706	ON340651	ON366605	ON568252
	DX38	ON453707	ON340653	ON366607	ON568253
	DX39	ON453734	ON340680	ON366634	ON568275
	DX40	-	ON340652	ON366606	-
	DX43	ON598591	ON340692	ON366646	-

	DX44	ON453742	ON340693	ON366647	-
	DX45	ON453720	ON340666	ON366620	-
	DX46	ON453728	ON340674	ON366628	ON568270
	DX47	ON453726	ON340672	ON366626	ON568268
	DX49	ON453695	ON340640	ON366594	ON568243
	DX50	ON453696	ON340641	ON366595	ON568244
	DX51	ON453694	ON340639	ON366593	-
	DX52	ON453731	ON340677	ON366631	-
	DX54	ON453697	ON340642	ON366596	ON568245
	DX55	ON453729	ON340675	ON366629	ON568271
	DX56	ON453698	ON340643	ON366597	-
	DX57	ON453715	ON340661	ON366615	ON568261
	DX58	ON453732	ON340678	ON366632	ON568273
	DX59	ON453690	ON340635	ON366589	-
	DX60	ON453733	ON340679	ON366633	ON568274
	DX61	ON453692	ON340637	ON366591	ON568241
	DX62	ON453719	ON340665	ON366619	-
	DX63	ON453716	ON340662	ON366616	-
	DX64	ON453701	ON340646	ON366600	ON568248
	DX65	ON453718	ON340664	ON366618	ON568263
	DX66	ON453730	ON340676	ON366630	ON568272
	DX70	ON598587	ON340684	ON366638	ON568278
	DX71	ON598590	ON340687	ON366641	ON568281
	DX72	ON598588	ON340685	ON366639	ON568279
	DX73	ON598589	ON340686	ON366640	ON568280
	DX74	ON453722	ON340668	ON366622	ON568265
	DX78	ON453735	ON340681	ON366635	ON568276
	DX79	ON453736	ON340682	ON366636	-
	DX81	ON453727	ON340673	ON366627	ON568269
	DX82	ON453717	ON340663	ON366617	ON568262
	DX84	ON453743	ON340694	ON366648	ON568286
Diaporthe phaseolorum	CBS 127465	-	MH877950	-	-
Diaporthe musigena	CBS 129519	-	MH876824	-	-
Phomopsis cuppatea	CBS 117499	-	MH874572	-	-
Nigrospora rubi	CGMCC 3.18326	-	KX986102	-	-
Nigrospora camelliae-sinensis	CGMCC 3.18125	-	KY806262	-	-
Valsella salicis	AR3514	-	AF408389	-	-

Table S3. Isolates *Colletotrichum* spp. studied and GenBank accession numbers of the generated sequences

Species	Accession number	GenBank accession			
		ITS	CAL	GAPDH	TUB2
C. aenigma	ICMP 18608	JX010244	JX009683	JX010044	JX010389
	CX12	ON329714	ON420249	ON394453	ON420396
	CX13	ON329715	ON420250	ON394454	ON420397
C. camellia	ICMP 10643, LF897, LC3667	JX010224	JX009630	JX009908	JX010436
	CX5	ON329717	ON420252	ON394456	ON420398
	CX6	ON329718	ON420253	ON394457	ON420399
	CX7	ON329719	ON420254	ON394458	ON420400
	CX8	ON329724	ON420259	ON394463	ON420405
	CX10	ON329720	ON420255	ON394459	ON420401
	CX25	ON329727	ON420262	ON394466	ON420408
	CX32	ON329723	ON420258	ON394462	ON420404
	CX37	ON329721	ON420256	ON394460	ON420402
	CX38	ON329722	ON420257	ON394461	ON420403
	CX39	ON329725	ON420260	ON394464	ON420406
	CX47	ON329726	ON420261	ON394465	ON420407
C. fructicola	ICMP 18646, CBS 125397	JX010173	JX009674	JX010032	JX010409
	CX1	ON329690	ON420225	ON394429	ON420372
	CX2	ON329709	ON420244	ON394448	ON420391
	CX3	ON329692	ON420227	ON394431	ON420374
	CX4	ON329691	ON420226	ON394430	ON420373
	CX9	ON329710	ON420245	ON394449	ON420392
	CX11	ON329695	ON420230	ON394434	ON420377
	CX14	ON329703	ON420238	ON394442	ON420385
	CX15	ON329696	ON420231	ON394435	ON420378
	CX16	ON329729	-	ON394468	ON420410
	CX17	ON329731	-	ON394470	ON420412
	CX18	ON329711	ON420246	ON394450	ON420393
	CX19	ON329704	ON420239	ON394443	ON420386
	CX20	ON329697	ON420232	ON394436	ON420379
	CX21	ON329698	ON420233	ON394437	ON420380
	CX22	ON329699	ON420234	ON394438	ON420381
	CX23	ON329712	ON420247	ON394451	ON420394
	CX24	ON329700	ON420235	ON394439	ON420382
	CX26	ON329694	ON420229	ON394433	ON420376
	CX27	ON329713	ON420248	ON394452	ON420395
	CX28	ON329693	ON420228	ON394432	ON420375
	CX29	ON329706	ON420241	ON394445	ON420388
	CX30	ON329705	ON420240	ON394444	ON420387
	CX36	ON329708	ON420243	ON394447	ON420390

	CX40	ON329707	ON420242	ON394446	ON420389
	CX42	ON329701	ON420236	ON394440	ON420383
	CX46	ON329730	-	ON394469	ON420411
	CX48	ON329702	ON420237	ON394441	ON420384
C. gigasporum	CBS 125475	KF687723	KF687813	KF687836	KF687874
	CX34	ON329734	ON420266	ON394473	ON420414
C. henanense	LC2820	KM610182	KM610176	KM610178	KM610184
	CX31	ON329728	ON420263	ON394467	ON420409
C. karstii	CBS 132134	HM585409	HM582013	HM585391	HM585428
	CX45	ON329732	ON420264	ON394471	-
C. siamense	ICMP 18578*, CBS 130417	JX010171	FJ917505	JX009924	JX010404
	CX41	ON329716	ON420251	ON394455	-
C. tropicicola	L58, LC0598*	JN050240	JN050229	JN050223	JN050246
	CX33	ON329733	ON420265	ON394472	ON420413
C. xanthorroeae	ICMP 17903, CBS 127831	JX010261	JX009653	JX009927	JX010448

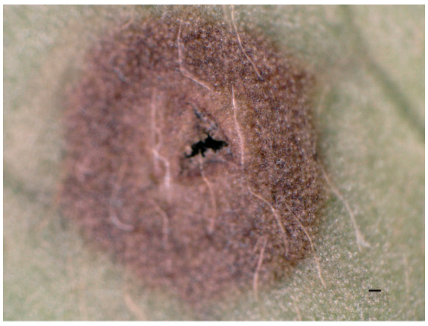

C

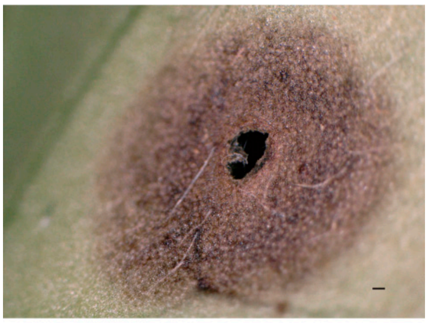

C+Dt

Figure S1. Details of lesions of inoculation with Cc and dual inoculations of Cc and Dt
